# Distribution and larval breeding habitats of *Aedes* mosquito species in residential areas of northwest Ethiopia

**DOI:** 10.4178/epih.e2018015

**Published:** 2018-04-23

**Authors:** Getachew Ferede, Moges Tiruneh, Ebba Abate, Wondmeneh Jemberie Kassa, Yitayih Wondimeneh, Demekech Damtie, Belay Tessema

**Affiliations:** 1 Department of Medical Microbiology, School of Biomedical and Laboratory Sciences, College of Medicine and Health Sciences, University of Gondar, Gondar, Ethiopia; 2 Ethiopian Public Health Institute, Addis Ababa, Ethiopia; 3 Department of Biology, College of Natural and Computational Sciences, University of Gondar, Gondar, Ethiopia; 4 Armauer Hansen Research Institute, Addis Ababa, Ethiopia

**Keywords:** *Aedes* mosquito, Breeding habitat, Risk indices, Arbovirus, Northwest Ethiopia

## Abstract

**OBJECTIVES:**

The *Aedes* mosquito is a vector for transmitting many arboviruses. Knowledge of the breeding habitat of this vector is vital for implementing appropriate interventions. Thus, this study was conducted to determine the breeding habitats and presence of *Aedes* mosquito species in the study areas.

**METHODS:**

A house-to-house cross-sectional survey of *Aedes* mosquito breeding habitats was carried out in Metema and Humera, Ethiopia, in August 2017. All available water-holding containers present in and around houses were inspected for the presence of immature stages of *Aedes* mosquitoes, and they were collected and reared to the adult stage for species identification. In the larval survey, the house index, container index, and Breteau index were computed as risk indices.

**RESULTS:**

Of the 384 houses surveyed for the presence of *Aedes* mosquito larval breeding, 98 were found to be positive for larvae. During the survey, a total of 566 containers were inspected, of which 186 were found to be infested with *Aedes* mosquito larvae, with a container index of 32.9, a house index of 25.5, and a Breteau index of 48.4. The most common *Aedes* mosquito breeding habitats were discarded tires (57.5%), followed by mud pots (30.0%). Of the 1,077 larvae and pupae collected and reared, *Aedes* *aegypti* (49.3%), *Ae. vittatus* (6.5%), and *Culex* species (44.2%) were identified.

**CONCLUSIONS:**

Discarded tires were the most preferred breeding habitats for *Aedes* mosquitoes. Moreover, *Ae. aegypti*, the main vector of dengue and other arboviruses, was identified for the first time in this region, suggesting a high potential for arbovirus transmission in the study areas.

## INTRODUCTION

The *Aedes* mosquito is responsible for the transmission of many arthropod-borne viruses (arboviruses), including dengue virus, yellow fever virus, Zika virus, and chikungunya virus [1]. These arboviruses pose increasing global public health concerns because of their rapid geographical spread and increasing disease burden. In particular, dengue is the most important arboviral disease, and is widely distributed in the tropical and sub-tropical regions of the world [2]. The problem of dengue has now extended to areas where it has not been reported earlier, including Ethiopia, where the first outbreak was reported in Dire Dawa and the Somalia region [3,4]. Moreover, the reemergence of yellow fever in Ethiopia after 50 years was also recently reported [5]. These diseases can only persist where their respective vectors (*Aedes* mosquitoes) are present. Several factors can facilitate the global expansion of these arboviruses, such as increasing uncontrolled urbanization and human population growth, the lack of proper waste management, and inadequate vector control measures [6]. Increased international travel and trade can move viruses and *Aedes* mosquitoes from one part of the globe to another, thus increasing arbovirus outbreak risks [7,8].

Infected female *Aedes* mosquitoes, mainly *Aedes aegypti* (Linnaeus) and also *Ae. albopictus* (Skuse), are the main vectors of several globally important arboviruses [9,10]. *Ae. aegypti* (Linnaeus) is currently distributed in urban areas and usually breeds in indoor and outdoor settings in a wide variety of natural and artificial water-holding containers such as plastic tanks, leaves, water storage jars, cement tanks, flower vases, curing tanks, glasses, rubber tires, and plastic bottles. Breeding habitats in urban areas arise mostly from neglected areas of construction sites and stagnant water that can create favorable conditions for mosquitoes to breed [11,12]. The destruction of *Aedes* mosquitoes breeding habitats reduces larval development, as well as the adult mosquito population and arbovirus transmission.

Most arboviral disease outbreaks occur during the rainy season and are associated with environmental factors such as rainfall, humidity, and temperature. These play a significant role in the transmission of arboviruses [13,14]. The likelihood of *Aedes* mosquitomediated outbreaks can be predicted by the use of risk indices such as the house index (HI), container index (CI), and Breteau index (BI) [12]. These indices are based on the simple determination of the presence or absence of *Aedes* mosquito larvae either in individual containers or somewhere in each house. These indices indicate the presence of *Aedes* mosquitoes and the potential risk of arboviruses and can be used to deploy appropriate interventions for the control of arboviral infections [15]. In particular, they can be used to support strategies for managing the population of *Aedes* mosquito larvae by releasing larvivorous predators into the collected water, eliminating breeding container habitats, and/or using insecticides [16]. Thus, knowledge of where *Aedes* mosquitoes breed is necessary for the implementation of effective control measures through larval control [17,18].

In countries neighboring Ethiopia, such as Sudan, Eritrea, Kenya, and Djibouti, various arboviral diseases have been reported [19-23]. Moreover, dengue outbreaks and yellow fever reemergence have recently been reported in Ethiopia [3,5]. Both towns included in the present study are on the border with Sudan, and many day laborers also migrate to these towns from different regions of Ethiopia in search of jobs linked with the large-scale farming of cash crops. Therefore, individuals infected with arboviruses such as dengue and yellow fever may disseminate the diseases in these towns, aided by the bites of *Aedes* mosquitoes. To control the emergence of such arboviral diseases, it is vital to control their respective vectors. However, there are no available data showing the presence or absence of *Aedes* mosquito species in the study areas. Therefore, this study aimed to provide the first baseline data on the presence of container-breeding *Aedes* mosquito larvae in various containers and to document the most predominant arbovirus vector (*Aedes* mosquito species) identified in the study areas. The evidence generated here will be vital for undertaking early prevention and control interventions.

## MATERIALS AND METHODS

### Study area, design, and period

A cross-sectional study was conducted in the towns of Metema and Humera in August 2017. Metema is located in northwest Ethiopia on the border with Sudan, 897 km north of Addis Ababa. This town has a latitude and a longitude of 12°58´N 36°12´E, with an elevation of 685 m above sea level. Humera is also located in northwest Ethiopia, 974 km from Addis Ababa, in the western zone of the Tigray Regional State, bordered on the west by Sudan and on the north by the Tekezé River, which separates Ethiopia from Eritrea. This town has a latitude and a longitude of 14°18´N36°37´E with an elevation of 602 m above sea level. Both study areas are among the most endemic areas for malaria in the country and are also among the most fertile agricultural zones, with large-scale farming of cash crops such as corn, sorghum, cotton, and sesame. In particular, the town of Metema serves as an important trade gateway between Sudan and the Amhara Region of Ethiopia.

### Data collection

**Figure 1.**
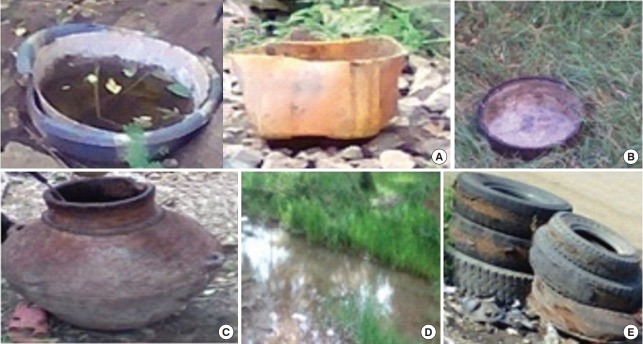
*Aedes* mosquito breeding habitats identified during a larval survey in the Ethiopian towns of Metema and Humera, (A) plastic containers, (B) mud dish, (C) mud pot, (D) ditch, and (E) discarded tires.

A house-to-house mosquito breeding habitat survey was conducted in both study areas. A total of 384 houses (179 in Metema and 205 in Humera) were included in the study. At the time of the study, Metema and Humera had 5,452 and 6,262 houses, respectively. The houses included in the study were selected by a systematic random sampling technique. From both towns, the first house was randomly included in the study. Thereafter, every 30th house was inspected for mosquito breeding in potential breeding habitats. The presence of immature stages of mosquitoes was visually evaluated in all water-holding containers present in indoor and outdoor areas and the premises of the houses (Figure 1). Outdoor areas were defined as the outside of the house, but confined to its immediate area (i.e., located within 10 m).

The number and type of containers inspected were recorded, including information on which had or did not have immature stages of mosquitoes. Larvae and pupae presumed to be *Aedes* mosquitoes were collected using a plastic cup, a pipette, or a ladle. The entire contents of the various containers were emptied into a large plastic tray or pan and the immature specimens were picked out using a dropper. They were placed immediately in labeled specimen bottles with water filled from a water container after collection and transported to an entomology laboratory. All collected larvae and pupae were reared in the laboratory until adults emerged, and all adults that emerged from the pupae were collected and stored in vials and carefully classified by species according to the pattern of white bands, using a dissecting microscope and identification keys [24,25].

### Data analysis

A descriptive analysis was conducted to calculate the proportions of various types of containers using Microsoft Excel 2007 (Microsoft Corp., Redmond, WA, USA). The risk indices HI, CI, and BI were calculated as follows:

(1) $$\mathrm{HI}\;=\;\frac{\mathrm{Number}\;\mathrm{of}\;\mathrm{houses}\;\mathrm{infested}}{\mathrm{Total}\;\mathrm{number}\;\mathrm{of}\;\mathrm{houses}\;\mathrm{inspected}}\times 100\;\mathrm{CI}\;=\;\frac{\mathrm{Number}\;\mathrm{of}\;\mathrm{positive}\;\mathrm{containers}}{\mathrm{Total}\;\mathrm{number}\;\mathrm{of}\;\mathrm{containers}\;\mathrm{inspected}}\times 100\;[26,27]\;\mathrm{BI}\;=\;\frac{\mathrm{Number}\;\mathrm{of}\;\mathrm{positive}\;\mathrm{containers}}{\mathrm{Total}\;\mathrm{number}\;\mathrm{of}\;\mathrm{houses}\;\mathrm{inspected}}\times 100$$

According to the Pan American Health Organization and the World Health Organization (WHO), an area is at a high risk of arbovirus transmission when these indices are above the threshold of 5% for the HI and BI, and 3% for the CI [28,29].

### Ethical clearance

The study was approved by the institutional review board of the University of Gondar. Informed consent was obtained from the owners/residents of each household prior to conducting the mosquito breeding site survey.

## RESULTS

### *Aedes* mosquito potential larval breeding habitats

**Figure 2.**
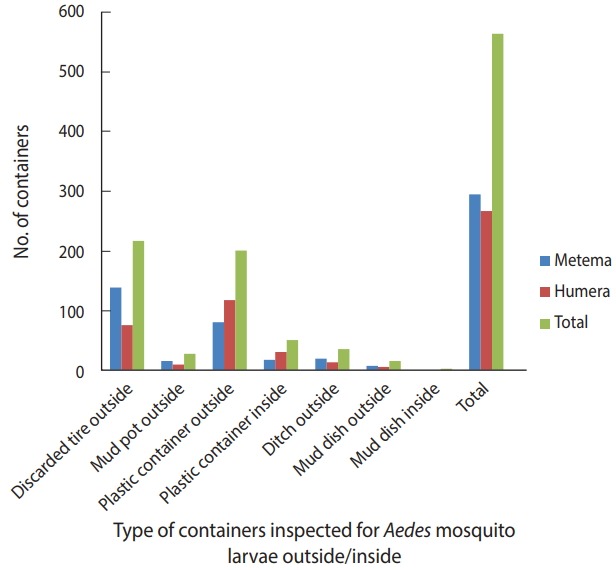
Water-holding containers inspected for *Aedes* mosquito larval breeding in the Ethiopian towns of Metema and Humera, August 2017.

**Figure 3.**
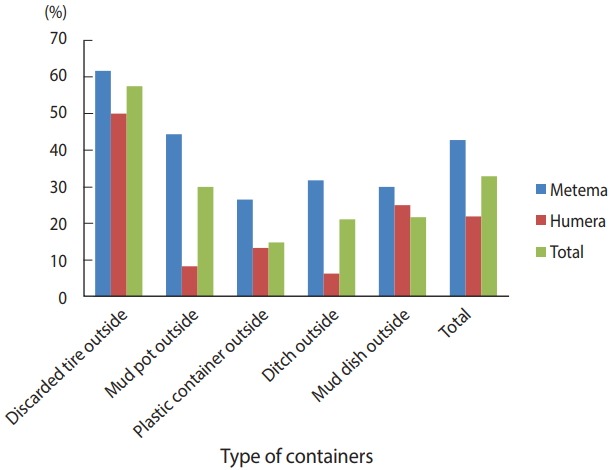
Distribution of containers infested with *Aedes* mosquito larvae in the Ethiopian towns of Metema and Humera, August 2017.

A total of 384 houses were surveyed, both outside and inside, in the towns of Metema and Humera to detect the presence of *Aedes* mosquito breeding sites. Overall, 566 water-holding containers were inspected during the survey (Figure 2), of which 186 (32.9%) were found to be infested with *Aedes* mosquito larvae. The type of water-holding container in the study with the highest rate of positivity for *Aedes* mosquito larvae was discarded tires (57.5%), followed by mud pots (30.0%), mud dishes (21.7%), ditches (21.1%), and plastic containers (14.8%). *Aedes* mosquito larval breeding was detected in all the listed types of water-holding containers that were found outside the homes (Figure 3).

### *Aedes* mosquito larval indices

**Figure 4.**
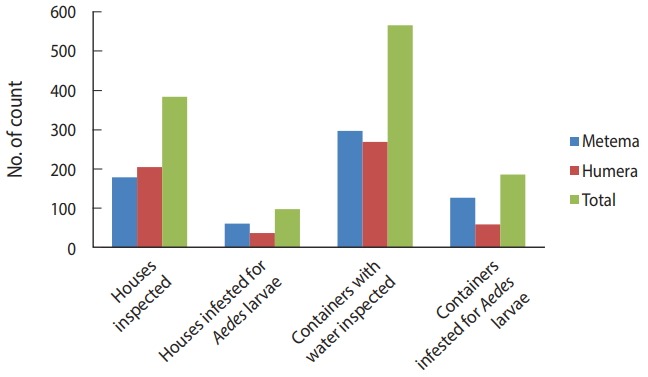
Houses and containers inspected for *Aedes* mosquito larval infestations in the Ethiopian towns of Metema and Humera, August 2017.

**Figure 5.**
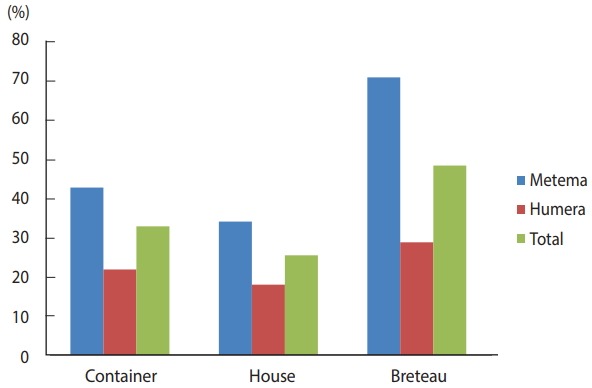
Container, house, and Breteau indices of *Aedes* mosquitoes in the Ethiopian towns of Metema and Humera, August 2017.

The BI, CI, and HI for *Aedes* mosquito larvae from both study areas were analyzed. Of the 384 houses surveyed, 98 had *Aedes* mosquito breeding habitats. The proportion of houses infested with *Aedes* mosquito larvae can be expressed as the HI, which was 25.5 in this study. Overall, 566 water-holding containers were identified, of which 186 had *Aedes* mosquito breeding. The CI was 32.9. The BI, which reflects the number of positive containers per 100 houses inspected, was recorded as 48.4. Regarding specific study areas, 61 of the 179 houses inspected in Metema had *Aedes* mosquito breeding (HI, 34.1) while 297 containers were inspected, of which 127 had *Aedes* mosquito breeding (CI, 42.8). In the town of Humera, 37 of the 205 houses inspected had *Aedes* mosquito breeding (HI, 18). In this area, 269 containers were identified as water-holding containers, of which 59 were found positive for *Aedes* mosquito breeding (CI, 21.9). In Metema and Humera, BIs of 70.9 and 28.8 were recorded, respectively (Figures 4 and 5).

### Species of adult *Aedes* mosquitoes identified

A total of 1,077 presumed *Aedes* mosquito larvae and pupae were collected from the 186 positive containers inspected and reared to the adult stage for species identification. Of these, 531 (49.3%) were *Ae. aegypti* (Linnaeus), 70 (6.5%) were *Ae. vittatus* (Bigot), and 476 (44.2%) were *Culex* species. *Ae. aegypti* (Linnaeus) and *Ae. vittatus* (Bigot) were identified in both Metema and Humera, suggesting a high risk of arbovirus transmission (Table 1).

## DISCUSSION

In this study, discarded tires had an especially high positivity rate for larvae of *Aedes* mosquitoes. This is consistent with other studies done elsewhere [30-32]. This might have been because the water collected inside tires is not easily observable. Discarded tires might also be stored for longer durations and harbor mosquito larvae undisturbed, making them prolific breeding containers [33]. Moreover, the weather conditions inside tires, such as cool temperature, humidity, and reduced light, create a suitable environment for *Aedes* mosquito breeding [14,34]. Eggs attached to the tires also play a role in the preservation of the *Aedes* mosquito population throughout the off season [35]. This study showed that *Aedes* mosquitoes seemed to breed in containers found outside the homes, not inside, which is in agreement with another study [36]. However, contrasting results were reported by other studies [37,38] that found that *Ae. aegypti* prefers to lay eggs and to rest indoors. A possible explanation for this discrepancy might be that many containers found outdoors are not mostly covered and filled with rainwater, and are therefore not ideal breeding sites for mosquitoes. These findings might have important implications for arbovirus vector control strategies, and in particular they may enable a more focused approach to vector control in which specific types of water-holding containers would be targeted. In such an approach, limited resources could be concentrated where they would have the greatest impact on disease transmission.

The presence of water-holding containers allows *Aedes* mosquito larvae to breed, thereby increasing the *Aedes* mosquito population and the concomitant risk for arbovirus transmission. In both areas in this study, the larval indices HI, CI, and BI, as indicated in Figure 5, were higher than the threshold values accepted by the Pan American Health Organization and WHO [28,29], but lower than those of a similar study in Dire Dawa, eastern Ethiopia in which the HI, CI, and BI were found to be 69.10, 54.00, and 134.55, respectively [39].The high observed values of the *Aedes* mosquito larval indices suggest a high risk of arbovirus transmission when arboviral cases become established in the area. Therefore, early interventions are necessary to combat the burden of emerging arboviral diseases.

In this study, the dominant *Aedes* mosquito species that emerged from the collected larvae was *Ae. aegypti*. This is in agreement with another study [39], and is also consistent with the preference of *Ae. aegypti* females to lay their eggs in domestic containers [40], but is in contrast with another study that identified *Ae. albopictus* as the most dominant species [41]. The presence of *Ae. aegypti* in this study is likely attributable to the abundance of suitable water-holding containers that are favorable for *Ae. aegypti* breeding and the availability of adequate organic material for its larval feeding [42]. This mosquito species is usually found in close proximity to human residences and feeds preferentially on human blood [43]. The strong preference for human blood exhibited by *Ae. aegypti* increases the potential for arbovirus transmission among humans. The second identified vector was *Ae. vittatus*, which is also a competent vector of arboviruses [44]. This *Aedes* mosquito species feeds on human and other animals, and this wide feeding behavior may somewhat limit its vectorial competency [45]. Future outbreaks of arboviral disease are possible in both areas included in this study due to the lack of native immunity in the population and the presence of *Ae. aegypti*, a major vector of arboviruses.

The study was the first attempt to characterize the presence of *Aedes* mosquitoes and their preferred breeding habitats in northwest Ethiopia. However, it has several limitations. The study was carried out during the rainy season, which might have led to high values of the risk indices. In all water-holding containers, the collected mosquito larvae and pupae were those presumed to be *Aedes* mosquito larvae and pupae; for this reason, the species of mosquito larvae and pupae could not be conclusively identified. Biotic and abiotic factors and water quality, which might affect the oviposition preferences of vector mosquitoes, were not checked, and this could be done in the future. Despite these limitations, importantly, this preliminary study provides the first baseline data on the presence of the arbovirus vectors *Aedes* mosquitoes in the study areas.

In conclusion, this study found that discarded tires were the most preferred *Aedes* mosquito breeding habitats, followed by mud pots. Moreover, the study documented the presence of *Ae. aegypti* and *Ae. vittatus* in the study areas for the first time, suggesting a high risk of arbovirus transmission. Therefore, the breeding containers identified should be subjected to appropriate control measures, such as source reduction via the removal of water-holding containers around living and working areas, and proper disposal of tires should be implemented.

## Figures and Tables

**Table 1.** Adult *Aedes* mosquito species identified from reared larvae and pupae in the Ethiopian towns of Metema and Humera, August 2017

Study area	Containers positive for larvae and pupae	Larvae and pupae collected	*Aedes aegypti*	*Aedes vittatus*	*Culex* species
Metema	127	760	409 (53.8)	25 (3.3)	326 (42.9)
Humera	59	317	122 (38.5)	45 (14.2)	150 (47.3)
Total	186	1,077	531 (49.3)	70 (6.5)	476 (44.2)

Values are presented as number or number (%).
